# Recovery of Low-Concentration Tungsten from Acidic Solution Using D318 Macroporous Resin

**DOI:** 10.3390/molecules29204946

**Published:** 2024-10-19

**Authors:** Xiangrong Zeng, Bin Zeng, Binjun Liang, Kuifang Zhang, Lijinhong Huang, Xinzhe Liu, Wanfu Huang

**Affiliations:** 1 College of Resources and Civil Engineering, Gannan University of Science and Technology, Ganzhou 341000, China; zengxr986@163.com (X.Z.); liangbinjun1205@163.com (B.L.); zhang_kui_fang@163.com (K.Z.); 2 Jiangxi Yaosheng Tungsten Industry Co., Ltd., Ganzhou 341000, China; 3 Jinyi Chuangdian (Tianjin) Technology Co., Ltd., Tianjin 300000, China; lxz19881101@163.com; 4 College of Resource and Environment Engineering, Jiangxi University of Science and Technology, Ganzhou 341000, China; angeline777@sina.com (L.H.); sim2008@sina.com (W.H.); 5 Faculty of Science and Engineering, WA School of Mines, Minerals, Energy and Chemical Engineering, Curtin University, Perth, WA 6152, Australia

**Keywords:** D318 macroporous resin, tungsten, adsorption capacity, desorption rate, kinetic analysis, thermodynamic analysis

## Abstract

Tungsten is a crucial strategic metal that plays a significant role in various fields, such as the defense industry, fine chemicals, and the preparation of new materials. During the practice of numerous tungsten smelting processes, a large amount of acidic wastewater containing low concentrations of WO_3_ is generated. The adsorption method, known for its simplicity, effectiveness, and ease of operation, represents the most promising approach for tungsten recovery and is vital for the sustainable development of the tungsten industry. In this study, D318 macroporous resin was used as an adsorbent to investigate its effectiveness in adsorbing WO_3_ from acidic solutions. Static adsorption experiments revealed that the adsorption capacity of D318 resin for WO_3_ was 683 mg·g^−1^. Kinetic analysis indicated that the controlling step for the adsorption of WO_3_ from acidic solutions by D318 resin was intraparticle diffusion. Thermodynamic analysis demonstrated that the adsorption process was endothermic and could occur spontaneously. By fitting the isothermal adsorption equation, it was found that the Langmuir model was more suitable for describing the adsorption process of WO_3_ on D318 resin in acidic solutions. The results of dynamic adsorption experiments showed that under optimized conditions, the dynamic adsorption capacity for WO_3_ was 529 mg·g^−1^; when using NaOH as the desorbent for cyclic desorption, the desorption rate for WO_3_ was 98.21%. XPS and SEM-EDS testing and analysis confirmed that D318 macroporous resin exhibited excellent adsorption performance for tungsten in acidic solutions.

## 1. Introduction

Tungsten is an important strategic metal that plays a significant role in the national defense industry, fine chemicals, new material preparation, and other fields [1,2]. Over the past two decades, significant progress has been made in the research and practice of tungsten smelting processes [3,4,5], resulting in a series of unique technologies such as sodium hydroxide decomposition followed by strong alkaline anion resin ion exchange [6], sodium carbonate decomposition with N263 extraction [7], sodium carbonate decomposition with N235 extraction, and sulfur–phosphorus mixed acid decomposition [8,9]. During the practice of numerous tungsten smelting processes [10,11], a large amount of wastewater containing low concentrations of WO_3_ is generated. This includes resin adsorption effluent, decomposition slag wash water, extraction raffinate [12], regenerant solutions, hydrochloric acid decomposition mother liquor, resin exchange column wash water, workshop floor wash water, etc., [13]. Typically, this wastewater contains WO_3_ concentrations ranging from 0.2 to 10 g·L^−1^, making it valuable for recovery. However, it also contains anions such as Cl^−^, CO_3_^2−^, and SO_4_^2−^, resulting in complex composition and variable properties. These characteristics lead to issues such as low processing efficiency, significant losses of WO_3_, high reagent consumption, and elevated treatment costs.

To efficiently and cost-effectively extract valuable metals from wastewater containing low concentrations of WO_3_, researchers have developed a resin adsorption method [14,15] for extraction, concentration, and purification [16]. Currently, researchers have primarily selected two types of resins for recovering tungsten from tungsten smelting wastewater containing low concentrations of WO_3_. The process of adsorbing tungsten using strongly basic anion resins is the most mature, with the 201 × 7 resin as a typical representative [17]. The advantage of this type of resin is that it completes the extraction, concentration, purification, and transformation of tungsten within a single system [18]. However, it also has issues such as poor adaptability, especially when the adsorbed solution contains high concentrations of anions such as Cl^−^, CO_3_^2−^, and SO_4_^2−^. In such cases, the adsorption capacity for WO_3_ is low, the adsorbed solution contains a high concentration of WO_3_, and the processing efficiency is poor.

The process of adsorbing tungsten using weakly basic macroporous anion resins, mainly represented by the D314 resin [16], has strong adaptability and good processing efficiency. Under acidic conditions, conventional anions do not affect their adsorption capacity for WO_3_, but there is a situation of relatively low adsorption capacity.

Based on this, Professor Zhao Zhongwei’s team developed the D301 adsorption process for WO_3_ in acidic solutions [19]. This process is capable of handling feed solutions with high concentrations of WO_3_, exhibiting a very high adsorption capacity for WO_3_ and demonstrating significant water-saving effects [20]. However, there is an issue that the concentration of WO_3_ in the solution remains relatively high after adsorption, necessitating secondary treatment. Therefore, it is not suitable for treating wastewater with low concentrations of WO_3_.

Based on the aforementioned context, this study employs D318 macroporous resin as the adsorbent to investigate its adsorption behavior towards WO_3_ in acidic solutions. Through kinetic and thermodynamic studies, the controlling processes of adsorption are analyzed. This research aims to provide a theoretical reference for the efficient and cost-effective recovery of low-concentration WO_3_ from wastewater.

## 2. Results and Discussion

### 2.1. Static Adsorption Kinetics

**Figure 1:**
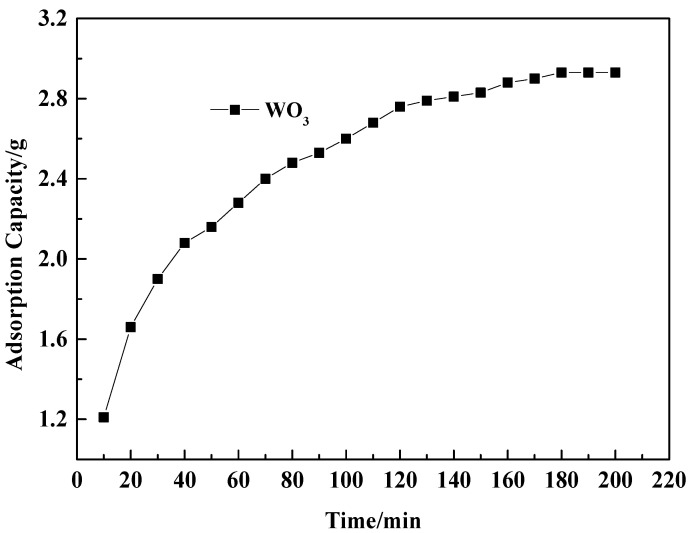
WO_3_ adsorption variation of adsorption time.

Study on static adsorption kinetics: A prepared 1000 mL acidic tungsten-containing solution (with a WO_3_ concentration of 7.79 g/L and a pH of 4.5) was placed in a glass beaker and stirred in a magnetic ion stirring water bath at a controlled speed of 120 r·min^−1^ and a temperature of 298 K. Subsequently, 5 g of pretreated resin was added to the glass beaker. For the experimental sampling, samples were periodically taken from the glass beaker using a pipette according to controlled conditions to detect the WO_3_ concentration in the samples and investigate the relationship between WO_3_ adsorption capacity and adsorption time. The test results are shown in Figure 1.

As can be seen from Figure 1, the adsorption capacity of D318 resin for WO_3_ in an acidic solution rises rapidly in the initial stage of adsorption and then gradually slows down. After the adsorption time reaches 180 min, the adsorption capacity for WO_3_ tends to stabilize, indicating that static adsorption equilibrium is achieved. Therefore, in subsequent static adsorption experiments, the adsorption time was controlled to be ≥180 min. When the static adsorption of WO_3_ by D318 resin reaches equilibrium, the equilibrium adsorption capacity is 586 mg·g^−1^.

**Figure 2:**
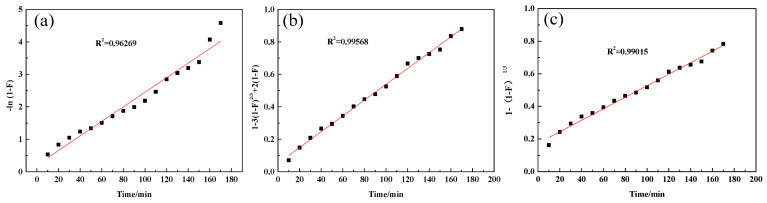
(**a**) Fitting curve of -ln(1-F) versus t. (**b**) Fitting curve of 1-3(1-F) ^(2/3)^ + 2(1-F) versus t. (**c**) Fitting curve of 1-(1-F) ^(1/3)^ versus t.

The adsorption process of D318 resin is an ion exchange process, which may be controlled by three steps: liquid film diffusion (-ln(1-F) = kt), intraparticle diffusion (1-3(1-F)^2/3^ + 2(1-F) = kt), and chemical reaction (1-(1-F)^1/3^ = kt) [21,22]. The step with the slowest reaction rate is the controlling step of the adsorption process. In the equations, F represents the adsorption degree, and k represents the adsorption rate constant at a temperature of 298 K. Based on the data from Figure 1 and the kinetic models, fitting curves were plotted, as shown in Figure 2.

As can be seen from Figure 2, 1-3(1-F)^2/3^ + 2(1-F) shows a better linear relationship with t, indicating that the controlling step for the adsorption of WO_3_ from acidic tungsten-containing solution by D318 resin is more likely to be intraparticle diffusion.

### 2.2. Static Adsorption Thermodynamics

#### 2.2.1. Effect of pH on Static Adsorption Capacity of WO_3_

**Figure 3:**
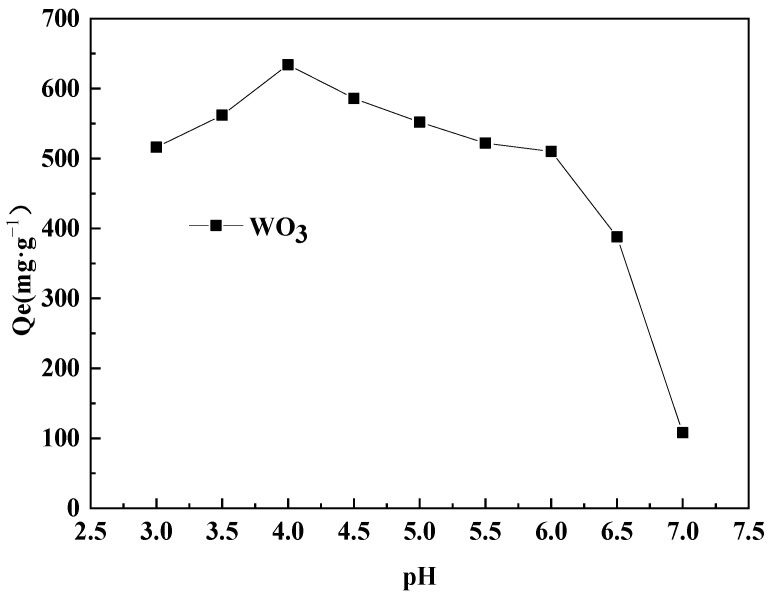
Effect of pH on adsorption.

In each experiment, 1000 mL of prepared acidic tungsten-containing solution with a WO_3_ concentration of 7.79 g·L^−1^ was used. The stirring speed was controlled at 120 r·min^−1^, the temperature was maintained at 298 K, and the adsorption time was set to 180 min. The influence of pH values of 3.0, 3.5, 4.0, 4.5, 5.0, 5.5, 6.0, 6.5, and 7.0 on the adsorption capacity of WO_3_ was investigated. The experimental results are shown in Figure 3.

As can be seen from Figure 3, the pH has a significant impact on the adsorption capacity of WO_3_ by D318 resin in an acidic solution. When the pH value gradually increases from 3.0 to 4.0, the adsorption capacity increases accordingly. After the pH exceeds 4.0, a further increase in pH leads to a decrease in adsorption capacity. At a pH of 7.0, the adsorption capacity drops sharply, indicating the occurrence of reverse reactions between adsorption and desorption. Therefore, controlling the pH of the acidic solution at 4.0 results in the best adsorption effect for WO_3_, with an adsorption capacity of 634 mg·g^−1^.

#### 2.2.2. Static Adsorption Isotherm

**Figure 4:**
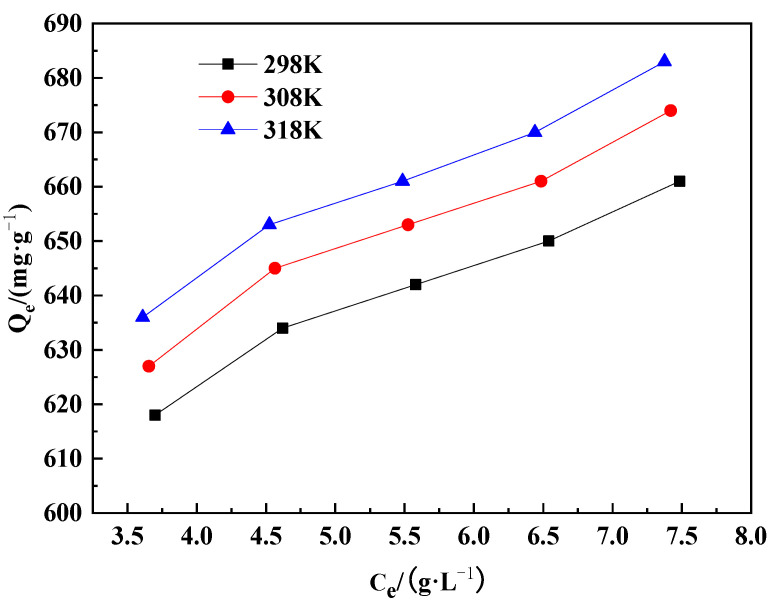
Equilibrium adsorption isotherms of WO_3_ on resin D318 at different temperatures.

In each experiment, 1000 mL of prepared acidic tungsten-containing solution with a pH value of 4.0 was used. The concentration of WO_3_ in the acidic solution was controlled at 6.79 g·L^−1^, 7.79 g·L^−1^, 8.79 g·L^−1^, 9.79 g·L^−1^, and 10.79 g·L^−1^, respectively. The stirring speed was maintained at 120 r·min^−1^, and the adsorption temperature was controlled at 298 K, 308 K, and 318 K, respectively, with an adsorption time of 180 min. Under these conditions, the adsorption isotherm of WO_3_ on D318 resin in an acidic solution was plotted, and the results are shown in Figure 4.

As can be seen from Figure 4, the adsorption capacity of WO_3_ on D318 resin in acidic solution increases slightly with temperature up to a maximum adsorption capacity of 683 mg·g^−1^. This increase is attributed to the facilitation of intraparticle diffusion by elevated temperatures. However, when the temperature exceeds 318 K, further increases in adsorption temperature can affect the stability and service life of the resin. Therefore, the operating temperature of the resin is controlled to not exceed 318 K.

To determine the adsorption behavior of WO_3_ in an acidic solution by D318 resin, the Langmuir and Freundlich isotherm equations were used for linear fitting of the isotherm equilibrium data. The equations used are as follows [23,24]:

Langmuir isotherm equation: (C_e_/Q_e_) = (C_e_/Q_m_) + 1/(K_L_Q_m_) (1)

**Figure 5:**
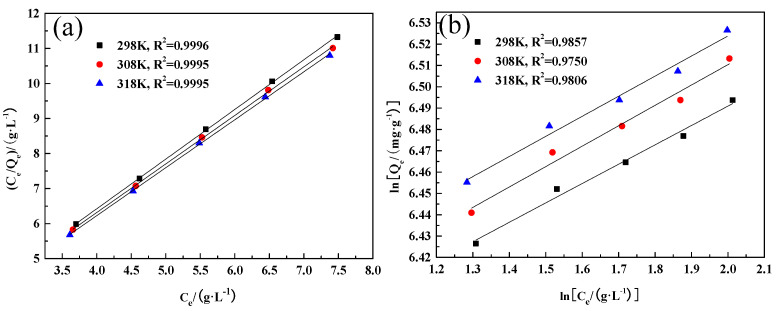
(**a**) Langmuir isotherm fitting curve; (**b**) Freundlich isotherm fitting curve.

Freundlich isotherm equation: ln(Q_e_) = ln(K_f_) + [ln(C_e_)]/n (2)

where C_e_ is the adsorption equilibrium concentration (g·L^−1^), Q_e_ is the equilibrium adsorption capacity (mg·g^−1^), and Q_m_, K_L_, K_f_, and n are constants for the Langmuir and Freundlich isotherm equations, respectively. Based on the experimental data, the fitting results are shown in Figure 5, and the relevant parameters calculated from the fitting data are presented in Table 1 and Table 2.

From Figure 5 and Table 1 and Table 2, the correlation coefficients for the isotherm equation fitting of the adsorption process of WO_3_ on D318 resin in acidic solution are obtained. The correlation coefficients for the Langmuir isotherm equation at 298 K, 308 K, and 318 K are 0.9996, 0.9995, and 0.9995, respectively, which are better than those for the Freundlich isotherm equation, indicating higher reliability. Therefore, the Langmuir isotherm equation is more suitable for describing the isothermal adsorption process of WO_3_ on D318 resin in an acidic solution. Calculations using the Langmuir isotherm equation reveal that the Q_m_ value is positive and increases slightly with temperature, indicating that elevated temperatures favor adsorption.

#### 2.2.3. Adsorption Thermodynamics

Thermodynamic data such as the enthalpy change during the adsorption of WO_3_ from acidic solution by D318 resin can be calculated using Equations (3) and (4) as follows:

ln(Q_e_/C_e_) = −ΔH/RT + ΔS/R (3)

ΔG = ΔH − TΔS(4)

where C_e_ is the equilibrium concentration (g·L^−1^), Q_e_ is the equilibrium adsorption capacity (mg·g^−1^), R is the constant with a value of 8.314 J·K^−1^·mol^−1^, T is the absolute temperature (K), ΔH is the enthalpy change in adsorption (KJ·mol^−1^), ΔS is the entropy change in adsorption (J·mol^−1^·K^−1^), and ΔG is the Gibbs free energy of adsorption reaction (KJ·mol^−1^). A plot of ln(Q_e_/C_e_) against 1/T × 103 was constructed, and through data fitting, the fitted equation ln(Q_e_/C_e_) = −0.252 × (1/T × 103) + 5.966 was obtained. After calculation, the thermodynamic data for the adsorption of WO_3_ from an acidic solution by D318 resin are presented in Table 3.

As can be seen from Table 3, the enthalpy change in adsorption ΔH is positive at adsorption temperatures of 298 K, 308 K, and 318 K, indicating that the adsorption process is endothermic. Therefore, appropriately increasing the temperature during the adsorption process is beneficial for the progress of the adsorption reaction. The Gibbs free energy of the adsorption reaction is negative at all these temperatures, indicating that the adsorption reaction can proceed spontaneously at 298 K, 308 K, and 318 K.

### 2.3. Dynamic Adsorption Experiment

#### 2.3.1. Effect of WO_3_ Concentration in Acidic Solution on Dynamic Adsorption Capacity

**Figure 6:**
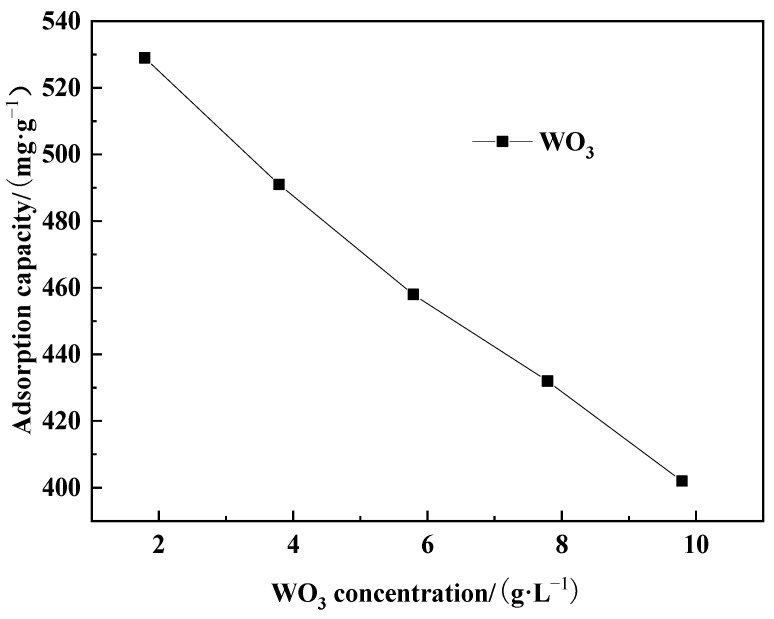
Feed liquid WO_3_ concentration variation of adsorption capacity.

For each trial, 260 g of pretreated D318 resin was weighed and added into a simulated exchange column with dimensions of φ25 mm × 700 mm. Subsequently, acidic solutions containing different concentrations of WO_3_ were pumped into the simulated column using a peristaltic pump for dynamic adsorption. The adsorption flow rate was controlled at 15 mL·min^−1^, the pH of the solution was maintained at 4.0, and the temperature of the solution was kept at room temperature. The endpoint of adsorption was determined when the concentration of WO_3_ in the effluent reached ≤0.05 g·L^−1^. The experimental results are shown in Figure 6.

As can be seen from Figure 6, when the concentration of WO_3_ in the effluent was controlled at ≤0.05 g·L^−1^ as the endpoint of adsorption, the concentration of WO_3_ in the acidic adsorption solution had a significant impact on the adsorption capacity. As the concentration of WO_3_ in the acidic solution increased, the overall adsorption capacity showed a downward trend. This is because, under the same controlled adsorption flow rate, a higher concentration of WO_3_ in the acidic solution made it more prone to leakage, leading to a decrease in adsorption capacity. Therefore, it can be inferred that D318 resin is more suitable for treating acidic solutions containing low concentrations of WO_3_. When the concentration of WO_3_ in the acidic solution was 1.79 g·L^−1^, the dynamic adsorption capacity was 529 mg·g^−1^.

#### 2.3.2. Effect of Adsorption Flow Rate on Dynamic Adsorption Capacity

**Figure 7:**
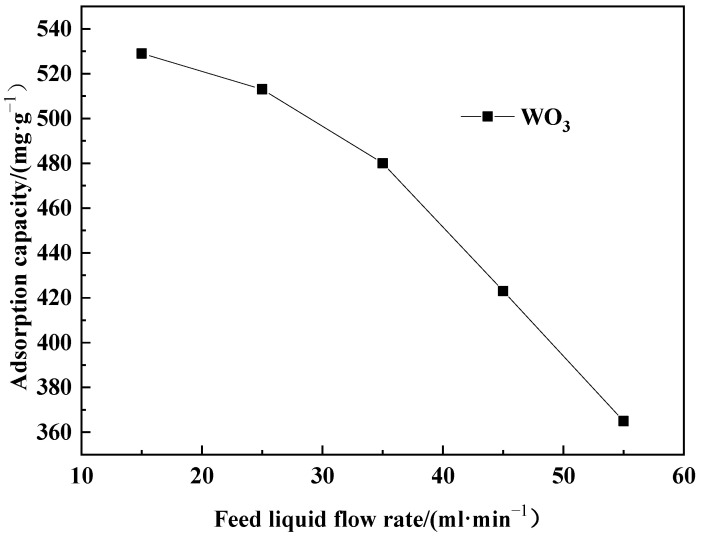
Feed liquid flow rate variation of adsorption capacity.

For each trial, 260 g of pretreated D318 resin was weighed and added into a simulated exchange column with dimensions of φ25 mm × 700 mm. Subsequently, an acidic solution containing WO_3_ (with a WO_3_ concentration of 1.79 g·L^−1^ and a pH value of 4.0) was prepared and pumped into the simulated column using a peristaltic pump for dynamic adsorption. The adsorption flow rates were controlled at 15 mL·min^−1^, 25 mL·min^−1^, 35 mL·min^−1^, and 45 mL·min^−1^, respectively, with the pH of the solution maintained at 4.0. The endpoint of adsorption was determined when the concentration of WO_3_ in the effluent reached ≤0.05 g·L^−1^. The experimental results are shown in Figure 7.

As can be seen from Figure 7, as the adsorption flow rate increases, the adsorption capacity decreases. A higher adsorption flow rate results in a shorter contact time between the solution and the resin bed. According to adsorption kinetics analysis, the adsorption process is controlled by intraparticle diffusion. A shorter contact time weakens intraparticle diffusion, leading to leakage and a reduction in adsorption capacity. Therefore, on the basis of balancing adsorption efficiency, keeping the adsorption flow rate as low as possible can enhance the adsorption capacity.

### 2.4. Dynamic Desorption Experiment

Experiment Preparation: Weigh 3380 g of pretreated D318 resin and subject it to static adsorption of an acidic solution containing WO_3_. After adsorption, filter and wash the resin then measure the concentrations of WO_3_ in the post-adsorption solution and the wash water. Calculate the adsorption capacity of the D318 resin for WO_3_ to be 512 mg·g^−1^. Divide the resin evenly into 13 portions (each equivalent to 260 g of dry resin) for use in the dynamic desorption experiment.

Desorption Experiment Method: Add the designated amount of resin into a simulated exchange column with dimensions of φ25 mm × 700 mm. Then, pump 650 mL of the prepared desorption agent into the simulated column using a peristaltic pump for dynamic desorption. Control the desorption flow rate according to the experimental design. From the start of desorption, collect the desorption solution, take samples every 5 min, measure the volume, and determine the WO_3_ concentration. After desorption is complete, mix the collected desorption solution, measure the total volume, and determine the WO_3_ concentration. Following the collection of the desorption solution, wash the resin with 650 mL of pure water at the same flow rate as the desorption agent. After washing, collect the wash water, measure the volume, and determine the WO_3_ concentration. Calculate the total desorption rate based on the amount of WO_3_ metal in both the desorption solution and the wash water.

#### 2.4.1. Effect of Desorption Agent Flow Rate on WO_3_ Desorption Efficiency

**Figure 8:**
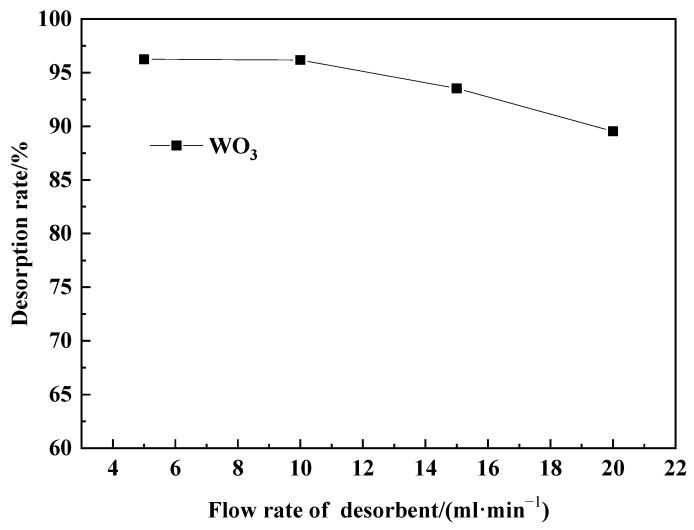
Flow rate of desorbent variation of desorption rate.

In each trial, prepared adsorbed resin (equivalent to 260 g of dry resin) was added into a simulated exchange column for dynamic desorption. The desorption agent volume was set at 650 mL, with a NaOH concentration of 84.74 g·L^−1^ (the amount of NaOH in the desorption agent was 1.2 times the theoretical dosage), and the wash water volume was 650 mL. The influence of desorption flow rates into the simulated exchange column, specifically 5 mL·min^−1^, 10 mL·min^−1^, 15 mL·min^−1^, and 20 mL·min^−1^, on the WO_3_ desorption efficiency was investigated. The results are shown in Figure 8.

As can be seen from Figure 8, a higher flow rate of the desorption agent results in a lower desorption efficiency of WO_3_. This is because a higher flow rate leads to a shorter contact time between the desorption agent and the loaded resin, resulting in insufficient desorption. When converting the flow rate of the desorption agent into the total time from the start of adding the desorption agent into the simulated exchange column until all of it is added, it can be observed that at desorption flow rates of 5 mL·min^−1^, 10 mL·min^−1^, 15 mL·min^−1^, and 20 mL·min^−1^, the contact time between the resin and the desorption agent is, respectively, 130 min, 65 min, 43.3 min, and 32.5 min (calculated based on the time when the resin at the top of the exchange column first contacts the desorption agent and the last time it contacts the desorption agent). Considering both desorption efficiency and desorption rate, the flow rate of the desorption agent can be controlled at 10 mL·g^−1^. At this flow rate, the contact time between the resin and the desorption agent is 65 min, and the desorption efficiency of WO_3_ is 96.18%.

#### 2.4.2. Effect of Desorption Agent Concentration on WO_3_ Desorption Efficiency

**Figure 9:**
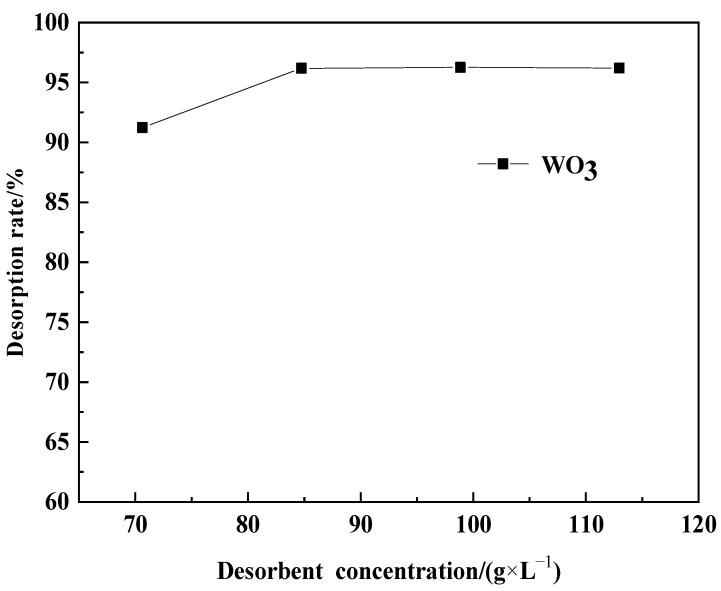
Desorbent concentration variation of desorption rate.

In each trial, prepared adsorbed resin (equivalent to 260 g of dry resin) was added into a simulated exchange column for dynamic desorption. The desorption agent volume was set at 650 mL, with a flow rate of 10 mL·min^−1^, and the wash water volume was 650 mL. The influence of NaOH concentrations in the desorption agent entering the simulated exchange column, specifically 70.62 g·L^−1^ (1 times the theoretical dosage), 84.74 g·L^−1^ (1.2 times the theoretical dosage), 98.86 g·L^−1^ (1.4 times the theoretical dosage), and 112.97 g·L^−1^ (1.6 times the theoretical dosage), on the WO_3_ desorption efficiency was investigated. The results are shown in Figure 9.

As can be seen from Figure 9, a higher concentration of NaOH in the desorption agent is beneficial for improving the desorption efficiency. After the NaOH concentration in the desorption agent reaches 84.74 g·L^−1^, further increasing the NaOH concentration results in only minor fluctuations in the WO_3_ desorption efficiency. Considering the cost of reagent consumption, the NaOH concentration in the desorption agent can be controlled at 84.74 g·L^−1^.

#### 2.4.3. Effect of Desorption Method on WO_3_ Desorption Efficiency

In each trial, prepared adsorbed resin (equivalent to 260 g of dry resin) was added to a simulated exchange column for dynamic desorption. The desorption agent volume was set at 650 mL, with a NaOH concentration of 84.74 g·L^−1^, and the wash water volume was 650 mL. The influence of different desorption methods on the WO_3_ desorption efficiency was investigated. For conventional desorption, the resin was added to the simulated column, followed by the injection of pure water and then the desorption agent. The flow rate of the desorption agent was controlled at 10 mL·min^−1^. After the addition of the desorption agent, 650 mL of pure water was further added to displace the desorption agent in the column. For dry desorption, the pure water in the simulated exchange column was drained, and then the desorption agent was added and allowed to soak for 30 min. Subsequently, the desorption solution was discharged, and the remaining desorption agent was added for another 35 min of soaking. The desorption solution was then discharged again, followed by washing with 650 mL of pure water. For cyclic desorption, the pure water in the simulated exchange column was drained, and then the desorption agent was added until the column was full. The desorption solution was gradually discharged while simultaneously pumping the discharged solution back to the top of the simulated exchange column. The cyclic flow rate of the desorption agent was controlled at 20 mL·L^−1^. After 65 min of cyclic desorption, the cycle was stopped, the desorption solution was discharged, and the column was washed with 650 mL of pure water. The experimental results are presented in Table 4.

As can be seen from Table 4, the cyclic desorption method resulted in higher WO_3_ desorption efficiency, a higher concentration of WO_3_ in the desorption solution, a lower concentration of WO_3_ in the wash water, and a higher direct recovery rate of WO_3_.

#### 2.4.4. Effect of Desorption Agent Type on WO_3_ Desorption Efficiency

In each trial, prepared adsorbed resin (equivalent to 260 g of dry resin) was added to a simulated exchange column for dynamic desorption. The desorption agent volume was set at 650 mL, with a wash water volume of 650 mL. Using the cyclic desorption method, the impact of two different desorption agents, NaOH solution (with a concentration of 84.74 g·L^−1^, 1.2 times the theoretical consumption) and ammonia water (with a free ammonia concentration of 74.15 g·L^−1^, 1.2 times the theoretical consumption), on the WO_3_ desorption efficiency was compared. The results are presented in Table 5. As can be seen from Table 5, when using NaOH solution at 1.2 times the theoretical amount as the desorption agent, a higher desorption efficiency of WO_3_ was achieved.

### 2.5. Analysis of Adsorption Mechanism

#### 2.5.1. SEM-EDS Test Analysis

**Figure 10:**
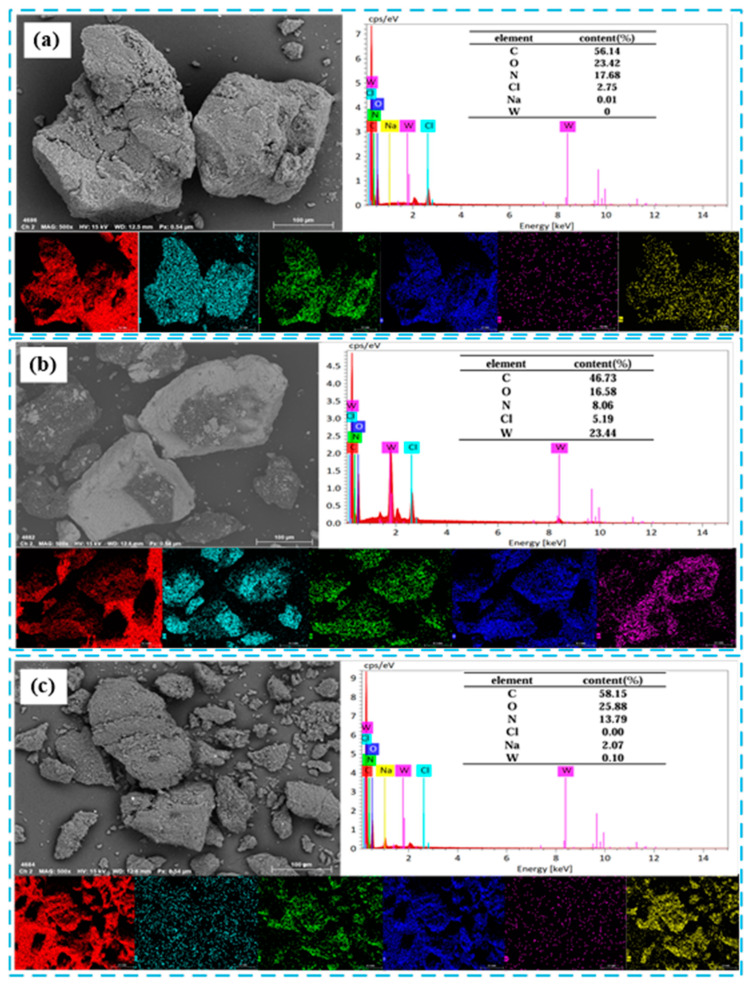
Morphology and element distribution of D318 resin before adsorption (**a**), after adsorption (**b**), and after desorption (**c**).

To verify whether the D318 macroporous resin successfully captured low-concentration tungsten from acidic solutions, SEM-EDS analysis was conducted on the morphology and element distribution of the D318 resin powder surface before adsorption, after adsorption, and after desorption. As evident from Figure 10, a significant amount of W element was detected on the surface of the D318 resin powder after adsorption compared to before adsorption, indicating that the D318 resin exhibits good adsorption efficiency for low-concentration tungsten in acidic solutions. After desorption, the tungsten content on the surface of the D318 resin was 0.1%, and the sodium content was 2.07%, demonstrating that sodium effectively desorbed tungsten from the resin, which is consistent with the experimental results.

#### 2.5.2. XPS Test Analysis

**Figure 11:**
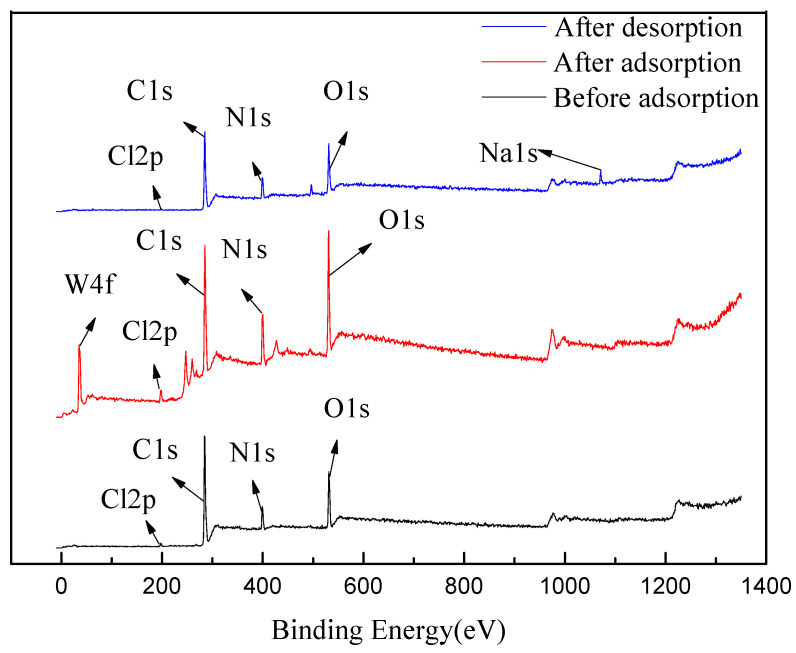
XPS before adsorption, after adsorption, and after desorption.

By analyzing the resin before adsorption, after adsorption, and after desorption using XPS, a more precise understanding of the adsorption behavior of tungsten in solution by D318 macroporous resin can be obtained. The results are presented in Figure 11 and Table 6. Compared with the resin before adsorption, the resin after adsorption showed a new peak of W4f, and the tungsten content increased from 0% to 2.61%, demonstrating the adsorption effect of D318 macroporous resin on low-concentration tungsten in acidic solution. When comparing the resin after desorption with the resin after adsorption, the W4f peak disappeared, with the tungsten content decreasing from 2.61% to 0%, while a new Na1s peak emerged, with its content increasing from 0% to 2.44%. This indicates that NaOH when used as the desorbent, exhibits excellent desorption efficiency for tungsten in D318 resin, which is consistent with the experimental results.

## 3. Experimental

### 3.1. Materials

The acidic solution containing WO_3_ required for the experiment was prepared by dissolving grade 0 ammonium paratungstate (APT) in an alkaline solution and then adjusting the pH with hydrochloric acid. The D318 resin used in the experiment was purchased from Wandong Hi-Tech (Tianchang) Co., Ltd. (Tianchang, China). It is a weakly basic macroporous anion exchange resin composed of a copolymer crosslinked polymer of methyl acrylate with a macroporous structure, containing 54–62% water, with a total exchange capacity (dry) of ≥7.2 mmol·g^−1^, a wet true density of 1.07–1.12 g·mL^−1^, a particle size range of 0.315–1.25 mm, a particle size of ≥95%, and a sphericity rate of ≥90%.

### 3.2. Resin Pretreatment

The procedure can be carried out as follows: Weigh the resin for the experiment. Soak the resin in pure water, ensuring the resin layer is fully submerged, for 48 h. Then, soak it in a 5% concentration of NaOH solution, ensuring the resin layer is fully submerged, for 24 h. Wash the resin with pure water, using 20 times the volume of pure water. Regenerate the resin by soaking it in a 3% dilute hydrochloric acid solution for 16 h, using 5 times the volume of the solution. Wash the regenerated resin with pure water until the pH of the effluent after washing is 5–6, indicating the completion of resin pretreatment. Filter, dry, weigh, and measure the volume of the pretreated resin, convert it into dry resin mass, and prepare it for subsequent experiments.

### 3.3. Static Adsorption Experiment

According to the experimental design, 1000 mL of prepared tungstic acid solution was placed in a 2000 mL glass beaker, which was then positioned in a magnetic ion stirring water bath. After the stirring speed and water bath temperature reached the control requirements, 5 g of pretreated resin was weighed and added to the glass beaker. For sampling, aliquots were periodically withdrawn from the beaker using a pipette according to the controlled conditions to detect the concentration of WO_3_ in the samples. The data were recorded, and the calculation formula is as follows [25].

The degree of adsorption of the target metal at a specific time in static condition:

F = Q_t1_/Q_e1_ = [V × (C_0_ − C_t_)]/[V × (C_0_ − C_e_)] (5)

where Q_t1_ represents the adsorption capacity of the target metal at a specific time (g), Q_e1_ represents the adsorption capacity of the target metal at equilibrium (g), C_t_ represents the concentration of the target metal in the adsorption solution at a specific time (g·L^−1^), C_0_ represents the initial concentration of the target metal in the adsorption solution (g·L^−1^), C_e_ represents the concentration of the target metal in the adsorption solution at equilibrium (g·L^−1^), and V represents the volume of the adsorption solution in the static adsorption experiment (mL).

### 3.4. Method for Dynamic Adsorption Experiments

For the adsorption test, the pretreated resin was weighed and added into a simulated exchange column. The prepared tungstic acid solution was then pumped into the column using a peristaltic pump for adsorption. After adsorption, the solution was collected quantitatively, numbered, and mixed for sampling, and the concentration of WO_3_ was measured. When the concentration of WO_3_ in the collected effluent from the simulated exchange column reached ≥0.05 g·L^−1^, the exchange was stopped. The residual solution in the simulated exchange column was washed with pure water and collected, and its volume was measured for the determination of WO3 concentration. The adsorbed metal amount of WO_3_ was then calculated.

For the desorption test, after washing, a desorption agent was pumped using a peristaltic pump, and the flow rate of the desorption agent was controlled. The desorption solution was collected, its volume was measured, and samples were taken for the determination of WO_3_ concentration. After the completion of desorption agent input, the column was washed with pure water, the wash flow rate was controlled, the wash water was collected, its volume was measured, samples were taken for the determination of WO_3_ concentration, and then the desorption rate was calculated. The calculation formulas are shown below.

Dynamic adsorption capacity for the target metal:

Q_d_ = Q_a_ − Q_b_ − Q_c_ = [(V·C_0_)/m] − [(V_1_·C_b_)/m] − [(V_2_·C_c_)/m] (6)

Dynamic desorption rate:

E_b_ = (V_3_ × C_d_)/(Q_d_ × m) (7)

where Q_d_ is the adsorption capacity (mg·g^−1^) when the concentration of WO_3_ in the effluent reaches a certain value, Q_a_ is the total capacity of WO_3_ entering the simulated exchange column (mg·g^−1^), m is the mass of D318 resin used in the experiment (g), C_0_ is the initial concentration of WO_3_ in the adsorption solution (g·L^−1^), C_b_ is the average concentration of WO_3_ in the effluent after adsorption (g·L^−1^), C_c_ is the average concentration of WO_3_ in the wash water from the simulated exchange column (g·L^−1^), C_d_ is the concentration of WO_3_ in the desorption solution (g·L^−1^), V is the total volume of adsorption solution entering the simulated exchange column (mL), V_1_ is the total volume of the effluent after adsorption (mL), V_2_ is the volume of wash water from the simulated exchange column (mL), and V_3_ is the volume of desorption solution.

## 4. Conclusions

This study focuses on the acidic solution of WO_3_, employing D318 macroporous resin for the adsorption and recovery of tungsten. The adsorption process is investigated through kinetic and thermodynamic analyses. Static adsorption experiments reveal that D318 resin adsorbs WO_3_ from acidic solutions under controlled conditions: a WO_3_ concentration of 10.79 g·L^−1^, pH 4.0, stirring speed of 120 r·min^−1^, adsorption temperature of 318 K, and adsorption time of 180 min, achieving an adsorption capacity of 683 mg·g^−1^ for WO_3_. Kinetic analysis indicates that the controlling step for D318 resin to adsorb WO_3_ from acidic solutions is intraparticle diffusion. Isothermal adsorption experiments suggest that the Langmuir isothermal equation is more suitable for describing the isothermal adsorption process of WO_3_ from acidic solutions by D318 resin. Thermodynamic analysis shows that the adsorption process is endothermic and can occur spontaneously and increasing the adsorption temperature can effectively enhance the adsorption capacity. Under optimized conditions, dynamic adsorption by D318 resin exhibits an adsorption capacity of 529 mg·g^−1^ for WO_3_, and dynamic desorption achieves a desorption rate of 98.21% for WO_3_. XPS and SEM-EDS testing and analysis align with the experimental results, demonstrating the effectiveness of D318 macroporous resin in adsorbing tungsten from acidic solutions. This work provides a cost-effective method for environmentally friendly recovery of low-concentration WO_3_ from acidic wastewater.

## Figures and Tables

**Table 1:** Fitting results of the Langmuir isotherm equation.

T/K	Fitted Equation	Qm	KL	R^2^
298	C_e_/Q_e_ = 1.41 Ce + 0.75	0.7206	1.41	0.9996
308	C_e_/Q_e_ = 1.38 Ce + 0.77	0.7424	1.38	0.9995
318	C_e_/Q_e_ = 1.36 Ce + 0.75	0.7543	1.36	0.9995

**Table 2:** Fitting results of the Freundlich isotherm equation.

T/K	Fitted Equation	n	Kf	R^2^
298	ln(Q_e_) = 0.090ln(Ce) + 6.309	0.1904	0.090	0.9857
308	ln(Q_e_) = 0.095ln(Ce) + 6.319	0.1912	0.095	0.9750
318	ln(Q_e_) = 0.094ln(Ce) + 6.335	0.1886	0.094	0.9806

**Table 3:** Results of adsorption thermodynamic data.

T/K	ΔG/(KJ·mol^−1^)	ΔH/(KJ·mol^−1^)	ΔS/(J·mol^−1^·K^−1^)
298	−12.686	2.095	49.601
308	−13.182		
318	−13.678		

**Table 4:** Effect of desorption methods on WO_3_ desorption rate.

Desorption Methods	Desorption Solution Volume/L	WO_3_ Concentration of Desorption Solution/(g·L^−1^)	Washing Solution Volume/L	WO_3_ Concentration of Washing Solution/(g·L^−1^)	Desorption Rate/%
Conventional desorption	0.645	184.29	0.615	14.89	96.18
Dry desorption	0.639	188.76	0.625	13.33	96.87
Cyclic desorption	0.642	191.31	0.615	12.87	98.21

**Table 5:** Effect of desorbent on WO_3_ desorption rate.

Desorbent	Desorption Solution Volume/L	WO_3_ Concentration of Desorption Solution/(g·L^−1^)	Washing Solution Volume/L	WO_3_ Concentration of Washing Solution/(g·L^−1^)	Desorption Rate/%
Sodium hydroxide solution	0.642	191.31	0.615	12.87	98.21
Ammonia solution	0.635	187.86	0.618	13.35	95.80

**Table 6:** Proportion of elements C, O, N, Cl, W, and Na in the sample.

	C1s	O1s	N1s	Cl2p	W4f	Na1s
Before adsorption	73.36	16.7	9.06	0.87	0	0
After adsorption	58.28	21.24	15.42	2.45	2.61	0
After desorption	69.16	16.89	10.79	0.71	0	2.44

## Data Availability

Data are contained within the article.
